# Identification of donors for low-nitrogen stress with maize lethal necrosis (MLN) tolerance for maize breeding in sub-Saharan Africa

**DOI:** 10.1007/s10681-019-2406-5

**Published:** 2019-03-28

**Authors:** Biswanath Das, Gary N. Atlin, Michael Olsen, Juan Burgueño, Amsal Tarekegne, Raman Babu, Eric N. Ndou, Kingstone Mashingaidze, Lieketso Moremoholo, Dickson Ligeyo, Rumbidzai Matemba-Mutasa, Mainassara Zaman-Allah, Felix San Vicente, B. M. Prasanna, Jill E. Cairns

**Affiliations:** 1 International Maize and Wheat Improvement Center (CIMMYT), United Nations Avenue, Gigiri, Village Market, PO Box 1041, Nairobi, 00621 Kenya; 2 CIMMYT, PO Box MP163, Harare, Zimbabwe; 3 Bill & Melinda Gates Foundation, PO Box 23350, Seattle, WA 98102 USA; 4 CIMMYT, Patancheru, India; 5 Agricultural Research Council-Grain Crop Institute, Private Bag X1251, Potchestroom, South Africa; 6 Kenya Agriculture and Livestock Research Organization, Kitale, Kenya; 7 CIMMYT, km. 35 Carr. Mexico-Veracruz, Texcoco, Edo. de Mexico, DF Mexico

**Keywords:** Maize, Low nitrogen-stress, Maize lethal necrosis, MLN, Breeding, Grain yield, Donor lines

## Abstract

After drought, a major challenge to smallholder farmers in sub-Saharan Africa is low-fertility soils with poor nitrogen (N)-supplying capacity. Many challenges in this region need to be overcome to create a viable fertilizer market. An intermediate solution is the development of maize varieties with an enhanced ability to take up or utilize N in severely depleted soils, and to more efficiently use the small amounts of N that farmers can supply to their crops. Over 400 elite inbred lines from seven maize breeding programs were screened to identify new sources of tolerance to low-N stress and maize lethal necrosis (MLN) for introgression into Africa-adapted elite germplasm. Lines with high levels of tolerance to both stresses were identified. Lines previously considered to be tolerant to low-N stress ranked in the bottom 10% under low-N confirming the need to replace these lines with new donors identified in this study. The lines that performed best under low-N yielded about 0. 5 Mg ha^−1^ (20%) more in testcross combinations than some widely used commercial parent lines such as CML442 and CML395. This is the first large scale study to identify maize inbred lines with tolerance to low-N stress and MLN in eastern and southern Africa.

**Electronic supplementary material:**

The online version of this article (10.1007/s10681-019-2406-5) contains supplementary material, which is available to authorized users.

## Introduction

Maize yields in sub-Saharan Africa (SSA) remain the lowest in the world, averaging 2 Mg ha^−1^, with production increases resulting largely from land expansion rather than higher yields per unit area (Cairns and Prasanna 2018). Low fertilizer use is one of the major contributing factors to continued low agricultural productivity in SSA relative to other regions (Fischer et al. 2014). Average fertilizer use in SSA was reported to be only 8 kg ha^−1^, compared to 100 and 96 kg ha^−1^ in Asia and Latin America respectively (Morris et al. 2007). At the Abuja Declaration on Fertilizer for the African Green Revolution in 2006 African Union Member States resolved to increase the level of fertilizer use to at least 50 kg ha^−1^ by 2015 (African Development Bank 2006). However, this deadline has passed and fertilizer use remains between 5 and 10 kg ha^−1^, far below the 50 kg ha^−1^ target set by the Abuja Declaration (FAOSTAT 2017). Thus most farmers in this region continue to produce maize in fields that are deficient in nitrogen (N). Fertilizers in SSA are amongst the most expensive in the world (Gregory and Bumb 2006) and many challenges need to be overcome to create a viable fertilizer market such as improved transport infrastructure and domestic production capacity (Crawford et al. 2006). The development and adoption of maize varieties with increased tolerance to low-N would provide an immediate intervention to moderately increase yields in smallholder farmers’ fields (Cairns et al. 2012a, b). For smallholder farmers in many parts of SSA low-N stress refers to maize grown with no fertilizer on severely N depleted soils resulting in yields between 1 and 2 Mg ha^−1^. There are very few breeding programs in the world that target this level of N stress intensity that is reflective of on-farm conditions in SSA (Bänziger et al. 2000). The genetic control of grain yield under optimal and low-N stress is partially independent (Ribaut et al. 2007), with the genetic correlation between grain yield under low- and high-N decreasing as yield under low-N decreases. Thus, direct selection for low-N tolerance is more efficient under low-N conditions where yields are at least 40% less than those of optimal conditions. If yields under low-N stress are greater than 60% of those in well-fertilised trials, selection is partly for genotypic yield potential rather than mechanisms of low-N stress tolerance, and N stress tolerant genotypes cannot be easily discriminated (Bänziger et al. 2000). The International Maize and Wheat Improvement Center (CIMMYT) began improving tropical and sub-tropical maize for low-N and drought stress tolerance in the 1970s and 1980s (Bänziger et al. 2006). Screening for low-N stress tolerance requires the development and maintenance of long-term sites depleted of N over several seasons to achieve target yield levels of 40% that of well-fertilised trials. As a result of this lengthy process that requires investment, only a limited number of nitrogen depleted sites (4 sites with a total area < 10 ha) were developed in eastern and southern Africa (ESA), where screening for drought stress remained the primary focus until 2009 (Magorokosho et al. 2010). In 2010, a large expansion of the CIMMYT-coordinated network of N-depleted yield testing sites was initiated in ESA in collaboration with national agricultural research systems (NARS) and private seed companies, in recognition of the importance of N-depleted environments in African maize production.

Maize lethal necrosis (MLN) is a viral disease of maize (*Zea mays* L.) caused by a combination of the maize cholorotic mottle virus (MCMV) and sugar cane mosaic virus (SCMV) or any other cereal virus of the potyviridae family. While SCMV had been reported in Kenya in 1980 (Louie 1980), MCMV and MLN are new in SSA. MLN was first reported in the Rift Valley of Kenya in 2011 (Wangai et al. 2012) and rapidly spread throughout East Africa (Adams et al. 2014; Lukanda et al. 2014; Mahuku et al. 2015; USDA 2014). In Kenya alone, production losses due to MLN in 2014 were estimated to be 10%, amounting to over 50 million USD (USDA 2014; De Groote et al. 2016). MLN infection resulted in almost complete yield loss in infected farmers’ fields (Wangai et al. 2012; Adams et al. 2014). The majority of commercially available varieties in East Africa and pre-release hybrids and elite inbred lines in eastern and southern (ESA) Africa showed high levels of susceptibility to MLN (Marenya et al. 2018). As the disease continues to spread it is crucial that new lines entering maize breeding pipelines in SSA have an acceptable degree of tolerance to MLN (Gowda et al. 2015).

Although genetic variability for low-N stress tolerance in tropical and sub-tropical maize has previously been reported (e.g. Akintoye et al. 1999; Presterl et al. 2003; Betrán et al. 2003; Worku et al. 2007), the number of lines, open pollinated varieties (OPVs) or hybrids used in each study was limited (less than 20). To date, no large-scale study has been conducted to identify the best tropical donors for low-N stress tolerance. The systematic characterisation of lines from a wide range of breeding programs allowed for the identification of the most tolerant drought donors in sub-tropical and tropical maize (Cairns et al. 2013a). These lines are now being used extensively in drought breeding worldwide. The aim of this study was to assess a diverse panel of elite tropical inbred lines from various maize breeding programs for tolerance to low-N stress. Given the rising importance of MLN in SSA, the panel was also evaluated in parallel for tolerance to MLN in order to identify key donors with tolerance to both low-N and MLN for deployment in maize breeding programs.

## Materials and methods

### Plant material

A collection of 431 elite, homozygous inbred lines was assembled from the Agricultural Research Council (ARC) in South Africa, the International Maize and Wheat Improvement Center (CIMMYT), and the Kenya Agriculture and Livestock Research Organization (KALRO) as shown on Table 1. The collection was assembled to identify low-N tolerant donors within active ARC, CIMMYT, and KALRO maize breeding programs. Information on the pedigree and adaptation zones of all lines is presented in Table S1. For the purposes of low-N screening in the target environment of ESA, all inbreds were test crossed to a common tester, CML539; a widely used tester throughout ESA with excellent general combining ability, adaptation and maize streak virus (MSV) tolerance. Due to seasonal variations in seed availability and shipment the number of testcrosses differed slightly in each experiment (Table 2).

**Table 1:** Summary of origin of maize lines within the panel

Breeding program	Focus of breeding program	No. of lines
ARC	Yield potential and disease tolerance for mid-elevation South African environments	99
CIMMYT-Genebank		42
CIMMYT-Kenya	Yield potential, drought and low nitrogen (N) tolerance,	20
CIMMYT-Lowland Tropics	Yield potential, insect resistance	72
CIMMYT-Physiology	Drought and low N tolerance	87
CIMMYT-Zimbabwe	Yield potential, drought and low N tolerance, maize streak virus (MSV) resistance	27
KALRO	Yield potential and disease tolerance for mid-elevation and highlands in Kenya	72

**Table 2:** Summary of trials conducted in Kenya, Mexico, South Africa, Zimbabwe and Zambia under low nitrogen (N) stress and optimal conditions

Location	Year/season	Country	Seasons of depletion	Soil properties (0–90 cm depth)		No. of entries	Density (plants m^2^)
				Soil nitrate—N (ppm)	Soil texture		
*Low N stress*							
Tlaltizapan	2010A	Mexico	2		Clay	184	6.67
Harare	2010B	Zimbabwe			Clay	347	5.30
Embu	2011A	Kenya	2	33	Clay	379	5.30
Kiboko	2011A	Kenya	5	11	Sandy clay loam	349	5.30
Kakamega	2011A	Kenya	2	11	Sandy loam	381	5.30
Cedara	2011B	South Africa	2		Sandy clay	327	5.30
Embu	2011B	Kenya	3	33	Clay	389	5.30
Harare	2011B	Zimbabwe	5		Clay	327	5.30
Kiboko	2011B	Kenya	6	11	Sandy clay loam	390	5.30
GART	2011B	Zambia	2		Sandy clay loam	346	5.30
Agua Fria	2012A	Mexico	8		Sandy loam	335	6.67
Embu	2012A	Kenya	4	33	Clay	304	5.30
Kiboko	2012A	Kenya	7	10	Sandy clay loam	390	5.30
*Optimal—highland*							
Kitale	2011A	Kenya	–	24	Sandy loam	381	5.30
Kakamega	2011A	Kenya	–	14	Sandy loam	381	5.30
Kitale	2011A	Kenya	–	24	Sandy loam	380	5.30
Kakamega	2012B	Kenya	–	14	Sandy loam	335	5.30
*Optimal—sub-tropical*							
Agua Fria	2011A	Mexico	–			346	6.67
Kiboko	2011A	Kenya	–	17	Sandy clay loam	347	5.30
Cedara	2011B	South Africa	–		Sandy clay	327	5.30
Kiboko	2012A	Kenya	–	17	Sandy clay loam	304	5.30
Kibos	2012A	Kenya	–	–	Clay	304	5.30

### Experimental conditions and measurements

(i) Low-N

Trials were conducted in ten locations across Kenya, Mexico, South Africa, Zambia and Zimbabwe (Table 3). Two treatments were used; optimal fertilization and low-N stress. The number of years of depletion at each location varied from two to six. Experiments were planted in one-row plots of 4 m length, with a final plant density of 6.67 plants m-2 (Mexico), 5.33 plants m-2 (Kenya, Zambia, Zimbabwe and South Africa). At all locations two seeds per hill were sown, then thinned to one seed per hill three weeks after emergence. An alpha-lattice design was used, replicated twice. In optimal trials all plots received 50 kg N ha^−1^, 100 kg P ha^−1^ and 50 kg K ha^−1^ as an NPK basal dressing at sowing. A second application of N (92 kg N ha^−1^) was applied as urea at the V6 stage. In low-N trials plots received P and K. All recommended plant, weed, and insect control measures were followed for both treatments.

**Table 3:** Summary of trial location coordinates, elevation and years experiments were conducted

Country	Location	Coordinates	Elevation (masl)	Years
Kenya	Kiboko	− 2.250, 37.730	990	2011, 2012
	Embu	− 0.500, 37.450	1492	2011, 12
	Kibos	− 0.070, 34.820	1184	2012
	Kitale	1.010, 35.000	1859	2011
	Kakamega	0.270, 34.740	1526	2011, 2012
Mexico	Agua Fria	20.530, − 97.430	90	2011, 2012
	Tlatizapan	18.680, − 99.130	940	2010
South Africa	Cedara	− 29.530, 30.280	1100	2011
Zambia	Golden Valley Agricultural Research Trust (GART)	− 14.170, 28.370	1173	2011
Zimbabwe	Harare	− 17.800, 31.050	1498	2010, 2011

Days to anthesis and silking were recorded when 50% of the plants had shed pollen and 50% of the plants had silks, respectively. The anthesis-silking interval (ASI) was calculated as days to silking— days to anthesis. At physiological maturity, plant height was measured on two representative plants per plot, then all plants were hand harvested and grain yield measured using an Almaco sheller. Grain weights were adjusted to 12.5% moisture content. Protein content was estimated using near-infrared reflectance spectroscopy (NIR) using the methodology described by Rosales et al. (2011).

(ii) MLN

All 431 inbred lines were evaluated per se twice at Narok (− 0.962, 35.385, 2835 masl) and once at Naivasha (− 0.640, 36.375, 1890 masl) in Kenya in 2013 and 2014. Experiments were planted in one-row plots of 4 m length, with a final plant density of 5.33 plants m^−2^. Two seeds per hill were sown, then thinned to one seed per hill 3 weeks after emergence. An alpha-lattice design was used, replicated twice. All recommended agronomic management practices were followed. Although both locations are MLN hotspots, the trials were artificially inoculated using the protocols described in Gowda et al. (2015) to ensure uniform disease incidence. Disease severity was scored on a plot basis using a 1–5 scale where 1 = no visible MLN symptoms, 2 = fine chlorotic streaks mostly on older leaves, 3 = chlorotic mottling throughout the plant, 4 = excessive chlorotic mottling on lower leaves and necrosis of newly emerging leaves (dead heart), and 5 = complete plant necrosis (Beyene et al. 2017). Disease ratings were taken twice (V12 and R1 growth stages) which provided the maximum range in disease response.

### Statistical analysis

Individual analysis for each environment, combination of location and year used a linear mixed model for an alpha lattice design with all effects considered as random except the replicate effect:

$$y_{ikl} = \mu + R_{k} + IB(R)_{lk} + G_{i} + e_{ikl}$$

where $\mu$ is the overall mean, $R_{k}$ is the effect of the *k*th replicate, $IB(R)_{lk}$ is the effect of the incomplete block within the *k*th replicate, *G*_*i*_ is the effect of the *i*th genotype, and $e_{ikl}$ is the experimental error.

Broad-sense heritability (*H*) was estimated as $H = \sigma_{g}^{2} /\left( { \sigma_{g}^{2} + \sigma_{e}^{2} /r} \right)$ where $\sigma_{g}^{2}$ is the genotypic variance and $\sigma_{e}^{2}$ is the residual variance. The divisor $r$ is the number of replicates.

Trials with yields of greater than 4 Mg ha^−1^ were not considered to have received significant low-N stress and were removed from the combined analysis. Combined analysis across environments was performed using the linear mixed model for the response variable $y$ as follow:

$$y_{ijkb} = \mu + E_{j} + R(E)_{kj} + IB(RE)_{lkj} + G_{i} + GE_{ij} + e_{ijkl}$$

where $\mu$ is the overall mean, *E*_*j*_ is the effect of the *j*th environment (location/year/management), $R(E)_{kj}$ is the effect of the *k*th replicate within the jth environment, $IB(RE)_{lkj}$ is the effect of the incomplete block within the *k*th replicate in the *j*th environment, *G*_*i*_ is the effect of the *i*th genotype, and *GE*_*ij*_ is the interaction effect of the *i*th genotype with the *j*th environment. All effects, except environment, were considered random. Broad-sense heritability (*H*) was estimated as $H = \sigma_{g}^{2} /\left( { \sigma_{g}^{2} + \sigma_{e}^{2} /re} \right)$ where $\sigma_{g}^{2}$ is the genotypic variance, $\sigma_{g \times e}^{2}$ is the genotype x environment and $\sigma_{e}^{2}$ is the combined across environments residual variance. The divisors $e$ and $r$ are the number of environments and the number of replicates per environment respectively.

Variance components were estimated by restricted maximum likelihood (REML) using the SAS procedure Mixed of SAS software V9.4 (SAS Institute Inc. 2015) to fit the models above, and Best Linear Unbiased Prediction (BLUPs) were computed for all genotypes and genotype by environment combinations.

The genetic correlations between environments were calculated using equations from Cooper et al. (1996) by

$$\rho_{g} = \frac{{\sigma_{{g(ii^{\prime } )}} }}{{\sigma_{g(i)} \sigma_{{g(i^{\prime } )}} }}$$

where $\sigma_{{g(ii^{\prime } )}}$ is the genotypic covariance between environments *i*th and *i’*th, and $\sigma_{g(i)}$ is the genotypic variance components of the environment $i$.

## Results

### Grain yield, phenology and plant height across environments

Under low-N stress average trial grain yields ranged from 1.60 to 4.60 Mg ha^−1^ (t ha^−1^) (Table 4). Grain yield under low-N stress was over 4 M ha^−1^ in Cedara (4.43 Mg ha^−1^) and Embu (4.26 and 4.60 Mg ha^−1^) and these sites were subsequently removed from further analysis. Under optimal conditions, average trial grain yields ranged from 4.53 to 8.95 Mg ha^−1^ in highland environments and 5.42–10.04 Mg ha^−1^ in sub-tropical environments. Under optimal conditions average grain yield combined across highland and sub-tropical environments was 7.43 Mg ha^−1^ and 7.95 Mg ha^−1^, respectively (Table 5). Combined across environments low-N stress reduced grain yield by approximately 65% compared to optimal controls in highland and sub-tropical environments. Low-N stress significantly reduced the number of ears per plant compared to the optimal conditions in highland and sub-tropical environments (*p* < 0.01). ASI was significantly higher under low-N stress compared to the optimal highland conditions (*p* < 0.01). Plant height was significantly reduced by 20% and 25% under low-N compared to optimal conditions in highland and sub-tropical environments, respectively. Low-N stress also significantly reduced ear height compared to optimal controls (*p* < 0.05). Protein content was also significantly reduced by 25% under low-N (*p* < 0.01).

**Table 4:** Summary of individual trials (BLUP, number of entries and *h*) under low nitrogen stress and optimal conditions (highlands and sub-tropical environments)

Year/season	Location	Number of entries	Grain yield (Mg ha^−1^)		Anthesis date (d)		Anthesis-silking interval		Plant height (cm)	
			Mean	*H*	Mean	*H*	Mean	*H*	Mean	*H*
*Low N stress*										
2010A	Tlaltizapan	184	2.69	0.64	58.4	0.86	3.78	0.62	167.4	0.57
2010B	Harare	347	1.67	0.16	77.5	0.66	2.93	0.36	143.5	0.36
2011A	Embu^a^	379	4.60	0.40						
	Kiboko	349	2.57	0.51	60.4	0.36	5.08	0.07	131.2	0.35
	Kakamega	381	2.24	0.40	88.8	0.54	0.79	0.12	167.7	0.57
2011B	Cedara^a^	327	4.43	0.28						
	Embu	389	3.71	0.37	70.6	0.79	1.46	0.25	173.2	0.71
	Harare	327	3.82	0.46	74.2	0.82	0.16	0.42	182.6	0.56
	Kiboko	390	2.93	0.47	70.0	0.87	3.90	0.67	118.2	0.49
	GART	346	1.60	0.44	66.2	0.73	3.63	0.42	158.5	0.31
2012A	Agua Fria	335	2.50	0.51	75.2	0.85	5.13	0.76	155.7	0.56
	Embu^a^	304	4.26	0.47						
	Kiboko	390	3.04	0.37	66.8	0.79	1.91	0.42	161.5	0.45
*Optimal—highland*										
2011A	Kitale	381	8.28	0.51	85.7	0.17	–	–	251.2	0.74
	Kakamega	381	7.86	0.55	81.7	0.74	–	–	202.3	0.46
	Kitale	380	8.95	0.69	88.9	0.03	–	–	246.7	0.71
2012B	Kakamega	335	4.53	0.55	81.9	0.32	–	–	183.6	0.67
*Optimal—sub-tropical*										
2011A	Agua Fria	346	5.42	0.72	78.1	0.83	–	–	210.9	0.41
	Kiboko	347	7.84	0.50	61.1	0.62	–	–	196.8	0.73
2011B	Cedara	327	7.61	0.50	60.5	0.53	–	–	262.3	0.17
2012A	Kiboko	304	10.04	0.70	62.0	0.92	–	–	226.0	0.79
	Kibos	304	9.09	0.58	61.9	0.87	–	–	234.5	0.81

^a^Removed from combined analysis

**Table 5:** Summary of combined analysis of trials under low N stress, and optimal trials at highland and sub-tropical (E3) locations using best linear unbiased prediction (BLUP)

Trait	Location								
	Low N			Optimal—highland			Optimal—sub-tropical		
	Mean	Range	H	Mean	Range	H	Mean	Range	H
Grain yield (Mg ha^−1^)	2.65	1.94–3.14	0.59	7.43	5.37–9.54	0.62	7.95	5.54–9.56	0.73
Ears per plant	0.87	0.82–0.92	0.42	0.98	0.96–1.02	0.10	1.04	0.93–1.28	0.61
Anthesis date (d)	62.3	64.2–75.0	0.94	66.6	62.8–70.4	0.75	85.5	85.2–85.7	0.06
ASI	2.43	1.07–3.95	0.70	1.03	0.21–2.93	0.45	–	–	–
Protein	7.89	7.43–8.62	0.52	10.54	9.90–11.56	0.48	–	–	–
Plant height (cm)	165.6	145.2–187.4	0.86	223.4	207.5–246.3	0.59	233.5	204.4–258.9	0.85
Ear height (cm)	73.7	61.3–91.1	0.85	110.4	98.7–126.3	0.53	103.8	83.7–129.4	0.84

### Tolerance to MLN

**Fig. 1:**
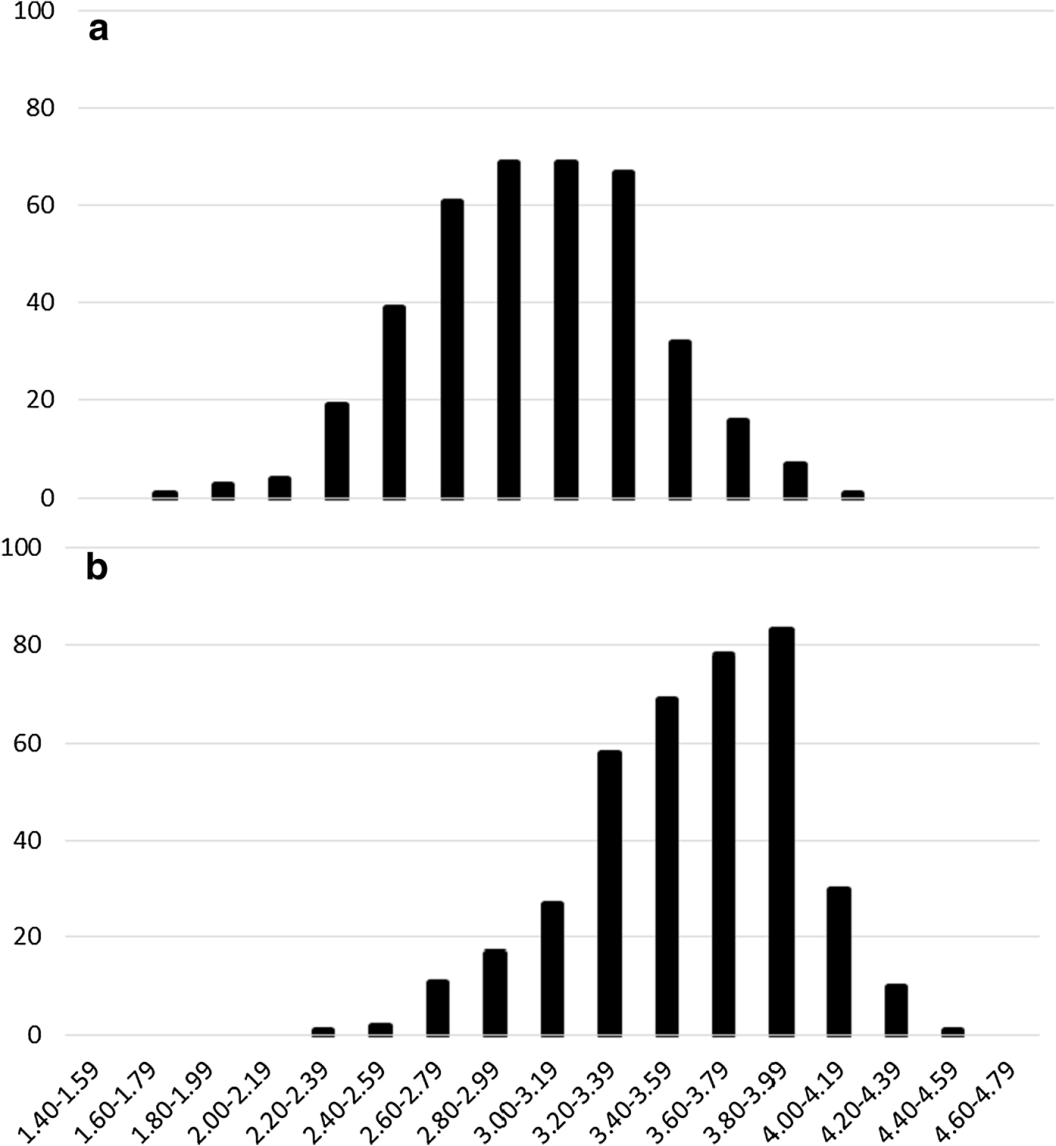
Genotypic variation in MLN disease score ratings at **a** V12 and **b** R1 growth stages using best linear unbiased predictions (BLUPs)

Average MLN disease scores at V12 and R1 were 2.98 and 3.57, respectively. All lines demonstrated some degree of susceptibility to MLN but large genotypic variability in response to MLN was observed (Fig. 1). Disease scores ranged from 1.74 to 4.14 at V12 and 2.17 to 4.45 at R1. H of MLN disease score at V12 and R1 growth stages was 0.71 and 0.67, respectively. Broad-sense heritability, variance components and genetic correlations between environments. In individual trials H of grain yield ranged from 0.16 to 0.64 under low-N stress, with 70% of the trials having an H of more than 0.40. Under optimal conditions H of grain yield ranged from 0.51 to 0.69 in highland environments to 0.50 to 0.72 in sub-tropical environments. Combined across 13 low-N environments H was 0.64. Combined across optimal environments H of grain yield was 0.62 in highland environments and 0.73 in sub-tropical environments. In individual trials H of ASI ranged from 0.12 to 0.67 under low-N stress. In 6 of the 10 low-N stress trials H of GY was higher than the H of ASI. In individual trials H of plant height ranged from 0.31 to 0.71 under low-N stress. Under optimal conditions H of plant height ranged from 0.46 to 0.74 in highland environments and 0.17–0.81 in sub-tropical environments. Combined across low-N environments H of plant height was 0.86. Combined across optimal environments H of plant height was 0.59 in highland environments and 0.85 in sub-tropical environments.

Under low-N stress the $\sigma_{g \times e}^{2}$ and $\sigma_{e}^{2}$ were higher relative to the $\sigma_{g}^{2}$ (Table 6). In all environments $\sigma_{e}^{2}$ accounted for the largest proportion of the phenotypic variance, however the pooled plot residual variance was only two to three times that of the genotypic variance under optimal conditions compared to nine times under low-N stress.

**Table 6:** Estimated variance components for grain yield form combined ANOVA across locations for low nitrogen, optimal (highlands) and optimal (sub-tropical). All estimates are calculated from standardized data

Variance components^a^	Low N	Optimal	
		Highland	Sub-tropical
$\sigma_{g}^{2}$	0.07	0.64	0.57
$\sigma_{g \times e}^{2}$	0.16	0.64	0.40
$\sigma_{e}^{2}$	0.63	1.87	1.27
No. of locations	10	4	5

^a^Where $\sigma_{e}^{2}$ is the environmental variance, $\sigma_{g}^{2}$ is the genotypic variance, $\sigma_{g \times e}^{2}$ is the genotype × environment and $\sigma_{e}^{2}$ is the residual variance

Grain yield under low-N stress was weakly negatively related to grain yield under optimal (highland conditions R^2^ = − 0.20, *p* < –  0.05). There was no significant relationship under optimal conditions and sub-tropical environments. In general, within individual locations, genetic correlations for grain yield for low-N were strongly, positively correlated between years. However the relationship between optimal trials in Kakamega was only weakly, positively correlated between seasons reflecting the bimodal rainfall climate in western Kenya.

### Key donors for low-nitrogen tolerance

The best 20 lines based on testcross performance under low-N are presented in Table 7 (grain yield BLUPs for all testcrosses under low-N stress and both optimal environments are also presented in Table S1). Common inbred lines, such as CIMMYT maize lines (CML) CML395 and CML442, yielded 0.4–1 Mg ha^−1^ less than the most tolerant lines and ranked in the bottom 50% for low-N tolerance. The average BLUP disease severity score for MLN was 3.0 with a range of 1.8–4.1. Although no line could be considered completely resistant to MLN, the top 4 lines (CLRCY039, DTPYC9-F46-1-2-1-2-B, CLRCY034 and CLWN270) scored less than 2 on the disease severity index and may be considered tolerant.

**Table 7:** Top 20 lines in combination with CML539 under low N stress. Best linear unbiased predictions (BLUPS) for grain yield under low N stress and optimal (highland and sub-tropical) conditions and disease score under MLN are presented

Entry	Pedigree	Heterotic group^a^	Colour	Low N	Optimal—highland		Optimal—sub-tropical		MLN	
				Grain yield	Grain yield		Grain yield			
				Mg ha^−1^	Mg ha^−1^	Rank	Mg ha^−1^	Rank	Disease score	Rank
64	CML341	AB	White	3.14	7.38	227	9.05	10	2.69	89
167	DTPWC9-F67-2-2-1-B	A	White	3.07	6.96	330	8.88	23	2.68	86
33	CZL068	B	White	3.06	7.51	186	7.78	223	3.55	362
193	LaPostaSeqC7-F64-2-6-2-2	A	White	3.03	7.29	248	8.27	122	2.69	97
31	CZL052	B	White	3.03			6.6	363	2.70	101
185	LaPostaSeqC7-F103-2-2-2-1	A	White	3.03	7.82	102	8.95	16	2.48	35
190	LaPostaSeqC7-F180-3-1-1-1	A	White	3.03	7.75	121	8.7	41	3.17	259
422	BSC-13	.	Yellow	3.01	7.82	103	8.03	170	2.37	22
66	CML343	AB	White	3.00	7.13	286	8.89	21	2.99	194
8	CKL05015	B	White	3.00	7.64	153	8.61	55	2.63	71
160	DTPWC9-F17-1-3-1-1	A	White	2.98	8.06	56	8.64	49	3.28	303
196	LaPostaSeqC7-F78-2-1-1-1	A	White	2.98	7.72	130	9.45	3	2.25	13
222	CLYN231	A	Orange	2.95	7.71	138	7.89	205	2.25	14
141	[MBR-ET(W)C1F139-2-1-B-2-B-B-B-B-B-BxMBRC5BcF13-3-1-2-B-B-B-B-1-2-B-B-BxCML264Q]-1-1	B	White	2.94	7.55	179	8.07	160	2.49	38
428	NAW 5867	.	White	2.94	7.03	309	9.53	2	2.72	106
171	DTPYC9-F13-2-3-1-2	A	Yellow	2.93	7.59	169	8.1	156	3.46	347
100	CLYN244	B	White	2.93	8.12	50	8.41	88	3.13	250
60	CML264	A	White	2.92	7.46	207	8.12	153	2.73	109
152	CML373	A	White	2.92	8.24	38	8.42	85	2.82	141
81	CML494	AB	White	2.92	8.05	60	8.93	19	2.21	9
*Common inbred lines within CIMMYT breeding in eastern and southern Africa*										
61	CML312	A	White	1.94	3.31	25	6.37	368	2.69	74
72	CML395	B	White	2.62	8.25	34	8.18	138	3.27	300
74	CML442	A	White	2.51	7.19	270	7.78	221	3.64	371
75	CML444	B	White	2.79	8.26	32	9.56	1	2.69	98
2	CML543	B	White	2.80	8.54	10	8.7	40	2.03	5
215	CML550	B	White	2.85	7.98	71	8.34	106	2.79	127
175	DTPYF46-1-2-1-2	A	Yellow	2.60	8.43	17	7.71	245	1.86	2
Trial mean				2.65	7.43		7.95		2.98	
LSD				0.69	1.99		1.49		0.98	

^a^Heterotic group assignment for breeding purposes within CIMMYT

## Discussion

As selection pressure increases for a specific trait, genetic variability inevitably decreases (Araus et al. 2018). To ensure progress new donors are required to bring new genetic variation. Screening a large number of elite, homozygous lines from a diverse, unadapted panel of lines across multiple locations and seasons in ESA allowed the identification of donor lines with stable tolerance to low-N stress for introgression into African adapted elite germplasm. Half of the lines originated from the La Posta Sequia (cycle 7) (LPS) and Drought Tolerant Populations (DTP) (cycle 9) developed in Mexico in the 1980s. CML341, CML343 and CML494 are also derived from La Posta Sequia recurrent selection populations (cycle 3 and 4) (CIMMYT 2005). The remaining lines were developed by four separate programs (CIMMYT Zimbabwe, CIMMYT Kenya, KALRO, CIMMYT tropical lowlands) that did not specifically target low-N tolerance during line development. The findings demonstrate both the importance of a targeted breeding approach for developing abiotic stress tolerant germplasm in addition to the value of germplasm exchange between global breeding programs with similar objectives. In this case, germplasm developed in tropical environments of Central America (CIMMYT Physiology and CIMMYT tropical lowlands program) demonstrated yield potential under both low-N stress and optimal conditions in ESA.

Five of the selected low-N tolerant donors (La Posta Sequia C7-F103-2-2-1, La Posta Sequia C7-F78-2-1-1-1, La Posta Sequia C7-F180-3-1-1-1, La Posta Sequia C7-F64-2-6-2-2 and [MBR-ET(W) C1 F139-2-1-B-2-B-B-B-BB-BxMBR C5 Bc F13-3-1-2-B-B-B-B-1-2-B-B-B × CML264Q]-1-1) were also previously identified as tolerant to drought, heat and combined drought and heat stresses (Cairns et al. 2013a). This study confirms tolerance to drought stress was related to tolerance to low-N stress (Bänziger et al. 1997, 1999, 2002). CIMMYT breeding programs have relied heavily on several key inbred lines (CIMMYT maize lines, CMLs) (Masuka et al. 2017a). An era study, comprised of 66 hybrids developed between 2000 and 2010, was recently assembled to evaluate genetic gain within the CIMMYT hybrid breeding program (Masuka et al. 2017a). Over half of these hybrids contained CML444 in their background, one-fifth contained CML312 and CML442. In this study, CML444 was relatively high under low-N stress while CML312, CML395 and CML442 ranked in the bottom 50% for low-N tolerance. Thus, it appears that substituting or improving these widely used inbred parents for low-N tolerance could potentially increase yields under low-N conditions by approximately 20%. MLN has devastated maize yields in Eastern Africa and has the potential to spread to other regions of SSA (De Groote et al. 2016). This is the first study to evaluate a wide array of tropical inbred lines (almost 400), including most key lines used in ESA breeding programs, for tolerance to MLN caused by MCMV and SCMV strains currently in East Africa. Several lines identified as tolerant to MLN such as CLRCY039, DTPYC9-F46-1-2-1-2-B, CLRCY034 and CLWN270 are yellow in grain colour and therefore need to be converted to either white or serve as donor lines in ESA where the preferred maize grain colour is white. Of the top 20 lines for MLN tolerance, over 50% were developed in Central America where MCMV has been reported since the late 1980s (Carrera-Martínez et al. 1989). A further 30% originated from the KALRO maize program in Western Kenya where there has been no history of MCMV until 2011. The results suggest that extensive genetic variation does exist for tolerance to the MLN causing viruses in East Africa amongst elite tropical inbred lines although issues with adaptation to target environments in ESA and consumer preferences (grain colour) need to be addressed. Extensive screening for MLN tolerance of all elite parental lines in African maize breeding program and publically available lines from other MCMV geographies should be undertaken to unearth further sources of tolerance.

Three of the lines with the highest grain yield under low-N stress were also in the top 5% for tolerance to MLN (La Posta Sequia C7-F78-2-1-1-1 (white), CLYN231 (yellow) and CML494 (white)). However, five of the top 20 best performing lines under low-N stress were highly susceptible to MLN (CZL068, La Posta Sequia C7 F180-3-1-1-1, DTPWC9-F17-1-3-1-1, CLYN244 and DTPYC9-F13-2-3-1-2), ranking in the bottom 40%. La Posta Sequia C7 F180-3-1-1-1 was previously identified as one of the best donors for drought stress breeding and has been extensively incorporated into maize breeding programs targeting stress prone environments (Cairns et al. 2013a). These results confirm the need to concurrently screen for the primary trait of interest and susceptibility to MLN to avoid new lines which are susceptible to MLN entering breeding programs in SSA.

### Implications for low-N stress breeding programs

Two lines ranked in the top 20 for low-N tolerance, optimal performance in sub-tropical environments and MLN tolerance: La Posta Sequia C7-F78-2-1-1-1-B and CML494. Both lines are white and are derived from La Posta Sequia recurrent selection populations developed in Mexico but showed good adaptation to screening environments in ESA. These lines should be prioritised for further improvement for key adaptive traits in ESA such as MSV and their introgression into maize breeding programs in ESA can play a considerable role in both addressing low-N stress and MLN. Bänziger et al. (1997) and Worku et al. (2007) previously found the relationship between grain yield under optimal and low-N stress to be positive, yet not high enough for grain yield under optimal conditions to be predictive of grain yield under low-N stress. In this study grain yield under low-N stress was negatively correlated to optimal conditions, although the relationship was weak. These results confirm the need to evaluate germplasm directly under low-N stress in order to develop new maize varieties for smallholder farmers in SSA. The low positive correlations reported previously and the low negative correlation reported here between yield under optimal and low-N conditions indicate that it is possible to select hybrids that are both tolerant to low-N conditions and responsive to fertilization. Four of the best performing low-N tolerant lines (CML341, La Posta Sequia C7-F103-2-2-2-1, La Posta Sequia C7-F78-2-1-1-1, and NAW5867) ranked amongst the top 20 lines for subtropical optimal performance suggesting they could potentially be used directly as parents in hybrid combinations to improve low-N tolerance. However the poor correlation between low-N and optimal performance suggests that most low-N tolerant donor lines will need to be introgressed into adapted, elite African germplasm to gradually improve low-N tolerance in the long term.

In almost two-thirds of trials H was higher for grain yield than ASI in contrast to earlier studies (Bolaños and Edmeades 1996; Cairns et al. 2012a, b). However these results are in agreement with recent large multi-location trials conducted in co-locating sites in ESA (Cairns et al. 2013a, b; Masuka et al. 2017a, b). The measurement of grain yield was mechanised in 2010 at most sites in this study and Cairns et al. (2013a, b) and Masuka et al. (2017a, b). ASI remains based on two visual scores of 50% anthesis and silking in each plot. We hypothesis that the higher H grain yield compared to ASI is, in part, a function of human error associated with visual and manual measurements. Visual and manual measurements are subjective, prone to human error, and lack robustness or repeatability (Araus et al. 2018; Gracia-Romero et al. 2018). Makanza et al. (2018) recently demonstrated a senescence index developed from images from a red–green–blue (RBG) camera mounted on an unmanned aerial vehicle (UAV) generally had higher heritability, and genetic correlations with grain yield, compared to visual measurements of senescence. Advances in remote sensing are likely to provide high-throughput and precise measurements of anthesis in maize in the very near future (Araus et al. 2018), however the development of tools to quantify silking, due to the location of maize ears, is likely to take longer. Low-N stress phenotyping sites should target a yield reduction of 60–75% relative to well-fertilised conditions at the same site (Masuka et al. 2012). To achieve this level of low-N stress requires significant investment to deplete the level of native soil N. Prior to 2009 capacity for low-N screening in Eastern and Southern Africa (ESA) was limited (< 10 ha) and the identification of low-N tolerant lines was confined to a few environments. Thirteen lines (CML504, CZL0212, CML258, CZL03004, CZL03018, CZL03009, CML442, CZL0617, CKL05009, CZL0619, CKL05006, (CML-395 × CL-RCW54)-B-14-1 and CLQRCWQ10) were previously considered to be tolerant to low-N stress yet ranked in the bottom 10% under low-N in the current study. The limited area for low-N screening previously limited all populations to be assessed for low-N stress tolerance and the lack of screening capacity islikely to be associated with the poor identification of key lines with tolerance. Recent studies of genetic gain in the CIMMYT ESA maize hybrid and OPV breeding programs showed gains under low-N stress were lower than under both managed and random drought stress within the hybrid breeding program (Masuka et al. 2017a, b). Genetic gain in hybrid grain yield under managed drought and random drought were both estimated at 0.85% per year, while gains under low-N stress were estimated at only 0.62% per year. These results highlight the need to place more emphasis on low-N breeding and selection in SSA, with large-scale screening under a range of locations with N depleted soils to identify robust lines with tolerance to N stress. The expansion of the drought breeding network in ESA facilitated the development of new DT maize germplasm with a 25% yield gain over commercial varieties in farmers’ fields within only 5 years (Cairns et al. 2013b; Setimela et al. 2017). Since 2010 an expanded low-N phenotyping network has been developed in Eastern and Southern Africa with sites in 10 countries amounting to over 48 hectares (120,000 plots) of low-N depleted area with almost 25% of the low-N network hosted by the private seed sector in southern Africa and 75% by national agricultural research systems. Depleting sites of low-N takes several years and the high yields at both Cedara and Embu reflects the age of these sites (less than 2 years of depletion). Making genetic gains for low-N tolerance breeding in ESA will depend to a large extent on maintenance of this extensive phenotyping platform which will require extensive continued regional and cross institutional collaboration (Zaman-Allah et al. 2015). Increased fertilizer use would be the most effective way to increase the productivity of small holder farmers in SSA, however fertilizer costs remain very high, largely due to high transportation and importation costs. Thus, in the near-term the development and adoption of maize varieties with increased tolerance to low-N stress can provide an immediate intervention to increase yields (Vergara-Díaz et al. 2016). Ideally, adoption of improved varieties should be complemented with improved agronomical practices that conserve and improve soil health in the long term. Introducing new technology to benefit subsistence farmers in this region requires careful consideration of the complexity of agricultural practices, the range of soil types and the culture of farming in regions where decreased crop yields on poor soil are a threat to food security. Nitrate is the dominant form of N in most agricultural soils and is highly soluble (Thorup-Kristensen et al. 2009) The N available for crop development originates from two main sources: mineralization of native soil organic matter, which is primarily influenced by the quality and quantity of organic resource inputs to soil, and applications of inorganic N fertilizers by farmers. Both sources are severely limited in many African soils. There is therefore a need to ensure that new low-N tolerant maize varieties don’t increase the rate of mining in depleted soils and are efficient in using the small amounts of mineral N fertilizer that smallholder farmers in SSA are likely to apply. Further work is required determine the mechanisms of nitrogen use efficiency in low-N tolerant donors lines.

## Conclusions

Genetic gain for low-N stress is currently lower than other abiotic stresses in Eastern and Southern Africa (Masuka et al. 2017a). To increase gains in maize yields under low-N stress increased emphasis needs to be placed on direct selection under low-N stress replicated in multiple locations in the target environment. This study identified lines that yielded about 20% more under low-N conditions than widely-used parental inbreds like CML442 and CML395 in test cross combinations with CML539. As MLN continues to spread throughout the region proactive approaches must be taken to ensure MLN susceptible lines are not used in breeding programs. Several of the most tolerant lines for low-N stress were susceptible to MLN, highlighting the need to screen all new donors for key stresses for tolerance to MLN prior to incorporation into breeding programs in SSA. Further work is required to understand the mechanisms of low-N stress tolerance and ensure low-N stress tolerant donors are more efficient at capturing nitrate that would be lost to the system rather than affecting the soil N-supplying capacity.

## Electronic supplementary material

Below is the link to the electronic supplementary material.

Supplementary material 1 (XLSX 52 kb)

## Abbreviations

ARC: Agricultural Research Council
ASI: Anthesis-silking interval
BLUP: Best linear unbiased prediction
CIMMYT: International Maize and Wheat Improvement Center
ESA: Eastern and southern Africa
GART: Golden Valley Research Trust
GS: Genomic selection
GWAS: Genome-wide association study
H: Broad-sense heritability
KALRO: Kenya Agriculture and Livestock Research Organization
$$\sigma_{e}^{2}$$: Error variance
$$\sigma_{g}^{2}$$: Genotypic variance
$\sigma_{g \times e}^{2}$: Genotype × environment variance
MLN: Maize lethal necrosis
MSV: Maize streak virus
N: Nitrogen
NARS: National agricultural research systems
OPV: Open pollinated variety
QTL: Quantitative trait loci
SSA: Sub-Saharan Africa
